# Mitochondrial pyruvate carrier: a potential target for diabetic nephropathy

**DOI:** 10.1186/s12882-020-01931-5

**Published:** 2020-07-14

**Authors:** Huanhuan Zhu, Huiting Wan, Lin Wu, Qing Li, Simeng Liu, Suyan Duan, Zhimin Huang, Chengning Zhang, Bo Zhang, Changying Xing, Yanggang Yuan

**Affiliations:** Department of Nephrology, The First Affiliated Hospital of Nanjing Medical University, Jiangsu Province Hospital, 300 Guangzhou Road, Nanjing, 210029 Jiangsu Province P. R. of China

**Keywords:** Mitochondrial pyruvate carrier, Mitochondria, Diabetic nephropathy

## Abstract

**Background:**

Mitochondrial dysfunction contributes to the pathogenesis of diabetic nephropathy (DN). Mitochondrial pyruvate carrier 1 (MPC1) and mitochondrial pyruvate carrier 2 (MPC2) play a bottleneck role in the transport of pyruvate into mitochondrial across the mitochondrial inner membrane. A previous study showed that increasing mitochondrial pyruvate carrier content might ameliorate diabetic kidney disease in db/db mice. However, the expression status of MPC1 and MPC2 in patients with DN is unclear.

**Methods:**

Patients with primary glomerulonephropathy (PGN, *n* = 30), PGN with diabetes mellitus (PGN-DM, n = 30) and diabetic nephropathy (DN, n = 30) were included. MPC1 and MPC2 protein levels were examined by immunohistochemistry. The expression of MPC in different groups was evaluated by the Kruskal-Wallis test. Spearman’s rank correlation was performed for correlation analysis between MPC levels and clinical factors.

**Results:**

Both MPC1 and MPC2 were localized in renal tubules. Levels of MPC1 and MPC2 were lower in DN patients than in PGN patients and in PGN patients with DM, whereas there were no differences in MPC1 and MPC2 levels among DN stage II to stage IV. Moreover, both MPC1 and MPC2 levels were significantly correlated with serum creatinine, BUN and eGFR in patients with DN, whereas no analogous trend was observed in nondiabetic kidney disease.

**Conclusions:**

Our study indicated that MPC localized in renal tubules, which were significantly decreased in DN. MPC was associated with clinical features, especially those representing renal functions.

## Background

Since the enormous increase in the global prevalence of diabetes mellitus (DM) over the past decades, diabetic nephropathy (DN) has transcended glomerulonephritis as the leading cause of the end-stage renal disease in many countries [1–3]. The pathogenesis of DN is complex and multifactorial. Of note, hyperglycemia is known to play a vital role in the development and progression of DN. The hyperglycemic state induces an increase in toxic glucose metabolites and mitochondrial dysfunction, and intensive glucose control could slow down the progression of DN [4, 5].

The kidney, as a highly metabolic organ, requires amounts of energy to maintain its normal function, which is intimately linked with mitochondria. Congruently, kidneys are rich in mitochondria and proximal tubules contain large numbers of mitochondria for synthesizing protein and reabsorbing metabolites [6, 7]. The reduction of mitochondrial content, increased mitochondrial DNA damage, and disruptions of mitochondrial networking (including fission and fusion) were observed in DN [6, 8–10]. Moreover, hyperglycemia increased the tricarboxylic acid cycle and altered the glycolytic pathway via the elevated level of advanced glycation end products, the activity of protein kinase C and hexosamine pathways and so on, which contributed to mitochondrial dysfunction [8, 11]. There were some investigations of patients and animal models have confirmed that mitochondrial dysfunction was postulated as a primary initiator and played a pivotal role in the progression of DN [12–15]. Hence, therapies that target mitochondrial function would be beneficial to alleviate DN progression, which has been verified by several mitochondria-targeted antioxidants including coenzyme Q10, mitoquinone, MTP-131 and so on [16–18].

Pyruvate, which is located at the branch point between oxidative and anaerobic metabolism, is critical for maintaining the stability of mitochondrial function [19]. Mitochondrial pyruvate carrier (MPC), a specific carrier in the inner mitochondrial membrane, imports pyruvate from the cytoplasm into the mitochondrial matrix. MPC is composed of two interactional subunits MPC1 and MPC2, and the expressions of both MPC proteins are essential for mitochondrial pyruvate transport [20–22]. MPC regulated mitochondrial substrate selection and was essential for several major pathways of carbohydrate, fat, and amino acid metabolism [23]. Depending on different organs, the MPC might play different effects on glucose homeostasis in type 2 diabetes. Hepatocyte-specific MPC disruption attenuated hyperglycemia in mice during high fat diet-induced obesity [24]. By contrary, MPC deficiency resulted in elevated blood glucose concentrations, glucose intolerance and reduced glucose-stimulated insulin secretion in pancreatic β-cells [25]. However, the role of MPC in kidneys of diabetes mellitus patients was still unknown. Therefore, we aimed to explore renal MPC expression in diabetic nephropathy.

## Methods

### Patients

A total of 30 biopsy-proven diabetic nephropathy (DN) patients were enrolled and 30 primary glomerulonephropathy (PGN) patients (membranous nephropathy, MN, *n* = 7; IgA nephropathy, IgAN, *n* = 6; focal segmental glomerulosclerosis, FSGS, *n* = 8; minimal change disease, MCD, *n* = 9) and 30 primary glomerulonephropathy patients with diabetes mellitus (PGN-DM) (MN, *n* = 11; IgAN, *n* = 8; FSGS, *n* = 7; MCD, *n* = 4) were used as controls. All the patients were admitted to the First Affiliated Hospital of Nanjing Medical University from September 2016 to May 2019. The exclusion criteria were as follows: (1) patients younger than 18 years old; (2) those obtained glomeruli were less than 5; (3) those with acute kidney injury; (4) those with severe organ insufficiency except kidney. The research complied with the Declaration of Helsinki and was approved by the ethical committees of the First Affiliated Hospital of Nanjing Medical University. Informed consent was obtained from each patient.

### Measurements

The following clinical data of patients were collected at the time of renal biopsy: age, gender, duration of diabetes, blood pressure, fasting glucose, glycosylated hemoglobin (HbA_1C_), blood urea nitrogen (BUN), serum creatinine, neutrophil gelatinase-associated lipocalin (NGAL), urine albumin, urine albumin-to-creatinine ratio (ACR) and so on. The urinary acidification function was measured using a ZDJ-4B automatic potentiometric titrator, which described in our previous study [26]. Estimated glomerular filtration rate (eGFR) was calculated according to the Chronic Kidney Disease Epidemiology Collaboration (CKD-EPI) formula [27].

Renal specimens were routinely examined by two experienced pathologists through light microscopy, immunofluorescence and electron microscopy. Diagnosis and classification of DN were made according to the criteria of Tervaert et al [28]. The glomerular classification was as follows: class I, glomerular basement membrane thickening and no specific; class IIa, mild mesangial expansion; class IIb, severe mesangial expansion; class III, nodular sclerosis; and class IV, global glomerulosclerosis in more than 50% of glomeruli. Diagnosis of MN, IgAN, FSGS, and MCD was made according to international standards.

### Immunohistochemistry

Paraffin sections were deparaffinized and rehydrated. Then, the sections were performed in citrate buffer (pH 6.0) for 10 min and washed with phosphate-buffered saline. After blocking by 5% bovine serum albumin for 1 h at room temperature, the sections were incubated with MPC1 antibody (1:900, Sigma, HPA045119) and MPC2 antibody (1: 20, Sigma, HPA056091) overnight at 4 °C. The sections were washed with PBS and then incubated with the second antibody for 1 h at 37 °C. The sections were then stained with 3, 3′-diaminobenzidine tetrahydrochloride for 10 min and counterstained with hematoxylin, dehydrated and mounted. Finally, each section was randomly captured 8–10 photographs by the microscope and calculated by the Image-Pro Plus.

### Statistical analysis

SPSS 22.0 software (SPSS, Chicago, IL, USA) was used for data analysis. Variables were expressed as the mean ± standard deviation. Comparisons for normal distribution groups were performed using the one-way analysis of variance. Differences among non-normal distribution groups were analyzed using the Kruskal-Wallis test. Correlations between MPC and clinical characteristics were assessed by using Spearman’s rank test. Significance was defined as *p* < 0.05.

## Results

### Patients characteristics

A total of 90 patients were included in this study and categorized into three groups according to the renal biopsy: those with biopsy-proven diabetic nephropathy (DN group, *n* = 30), those with biopsy-proven primary glomerulonephropathy (PGN group, *n* = 30), and those with biopsy-proven PGN and with diabetes mellitus (PGN-DM group, *n* = 30). The baseline characteristics of patients were shown in Table 1. Gender and age were comparable among groups. Of the DN patients, 6 (20%) were female, and their mean age was 50.57 ± 11.71 years, and the mean duration of diabetes was 9.94 ± 6.77 years. The estimated GFR was 59.64 ± 33.42 ml/min/1.73 m^2^ at baseline and proteinuria was 5.50 ± 5.10 g/24 h.

**Table 1:** Baseline characteristics in patients (*n* = 90)

Characteristics	PGN (*n* = 30)	PGN with DM (*n* = 30)	DN (n = 30)	*p* value
Female, n (%)	11 (36.7%)	10 (33.3%)	6 (20%)	0.274
Age (years)	47.17 ± 13.60	51.73 ± 14.06	50.57 ± 11.71	0.382
Diabetes duration (years)	0	3.77 ± 3.87	9.94 ± 6.77	< 0.001
Hypertension (years)	3.07 ± 3.94	3.77 ± 5.73	6.19 ± 7.45	0.105
Hypertension, n (%)	15 (50%)	16 (53.3%)	24 (80%)	0.033
SBP (mmHg)	133.73 ± 16.26	134.00 ± 17.66	146.00 ± 25.19	0.030
DBP (mmHg)	85.07 ± 11.72	84.00 ± 9.69	88.47 ± 13.48	0.310
Hb (g/L)	139.90 ± 14.11	137.40 ± 17.10	109.07 ± 22.51	< 0.001
HbA_1C_ (%)	5.61 ± 0.59	7.15 ± 1.30	7.57 ± 1.94	0.002
Fasting glucose (mmol/L)	4.94 ± 0.96	6.57 ± 1.84	8.20 ± 4.95	< 0.001
Total cholesterol (mg/dL)	6.47 ± 3.53	6.85 ± 2.78	5.66 ± 1.74	0.293
LDL cholesterol (mg/dL)	4.20 ± 2.39	4.46 ± 1.90	3.80 ± 1.30	0.180
HDL cholesterol (mg/dL)	1.34 ± 0.45	1.27 ± 0.41	1.16 ± 0.39	0.396
Triglyceride (mg/dL)	2.10 ± 1.51	3.40 ± 2.40	1.96 ± 1.84	0.004
Albumin (g/L)	32.93 ± 11.12	29.79 ± 9.67	32.24 ± 6.19	0.388
Serum creatinine (umol/L)	73.24 ± 21.51	84.65 ± 51.27	155.84 ± 95.87	< 0.001
eGFR (ml/min/1.73 m^2^)	100.10 ± 20.16	92.15 ± 27.99	59.64 ± 33.42	< 0.001
Serum cystatin C (mg/L)	1.07 ± 0.27	1.28 ± 0.51	2.05 ± 0.96	0.002
BUN (mmol/L)	5.44 ± 1.18	5.79 ± 2.06	10.63 ± 6.19	< 0.001
Uric acid (umol/L)	370.60 ± 87.13	387.97 ± 83.67	405.54 ± 103.96	0.344
Serum NGAL (ng/mL)	169.18 ± 96.86	238.54 ± 112.51	227.65 ± 149.28	0.053
Urine NGAL (ng/mL)	60.99 ± 116.73	95.43 ± 186.92	96.73 ± 146.77	0.394
Urine albumin(g/24 h)	3.44 ± 4.59	4.97 ± 4.62	5.50 ± 5.10	0.037
Urine ACR (mg/g)	119.71 ± 110.37	287.55 ± 146.89	465.32 ± 304.63	< 0.001
Urine pH	6.09 ± 0.57	5.89 ± 0.56	5.94 ± 0.66	0.398
Urine bicarbonate (mmol/L)	24.70 ± 2.42	24.63 ± 1.57	25.26 ± 2.21	0.324
Urine titratable acid (mmol/L)	24.03 ± 16.62	18.26 ± 10.12	12.59 ± 9.78	0.011
Urine ammonia (mmol/L)	44.67 ± 26.83	31.74 ± 18.89	18.48 ± 12.83	< 0.001

Diabetic nephropathy patients had longer diabetes duration (*p* < 0.001), higher levels of fasting glucose (*p* < 0.001) and HbA_1C_ (*p* = 0.002). The serum creatinine (*p* < 0.001), BUN (*p* < 0.001) and cystatin C (*p* = 0.002) levels were significantly higher in the DN group, whereas eGFR (*p* < 0.001) was lower. And there were also significant differences in urinary albumin (*p* = 0.037) and ACR (*p* < 0.001) among groups. Moreover, lower urinary titratable acid (*p* = 0.011) and ammonia (*p* < 0.001) were observed in the DN group. Therefore, serum fasting glucose, HbA_1C_, creatinine, BUN, cystatin C, eGFR, urinary albumin, ACR, urinary titratable acid, and urinary ammonia were parameters that were chosen for further analysis.

### MPC expression in the kidney

**Fig. 1:**
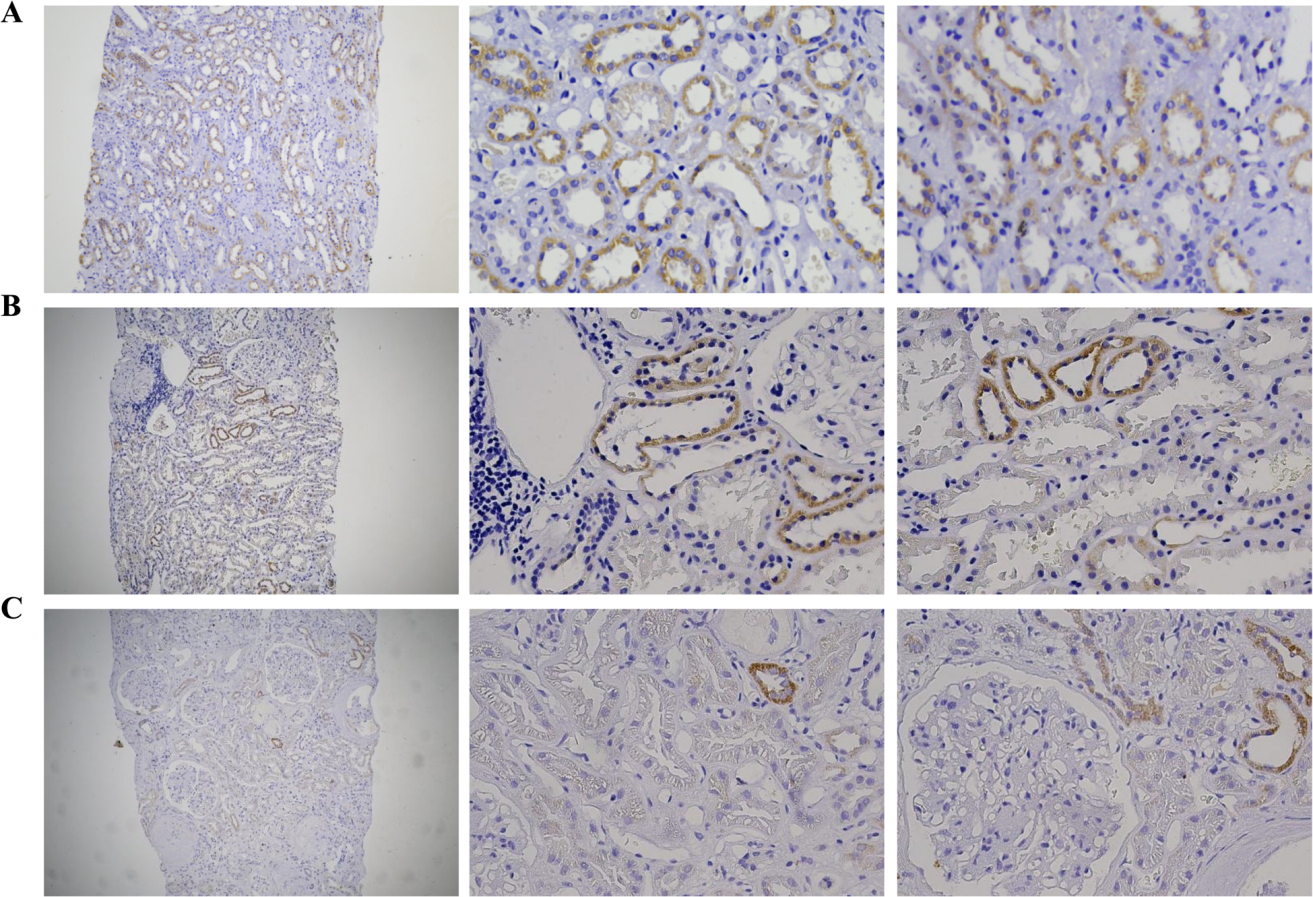
MPC1 expression in kidney tissues. Representative immunohistochemical staining of MPC1 in PGN (**a**, *n* = 30), PGN-DM (**b**, *n* = 30) and DN (**c**, *n* = 30) groups, respectively. Left column (Magnification× 100)*.* Right two columns (Magnification× 400)

**Fig. 2:**
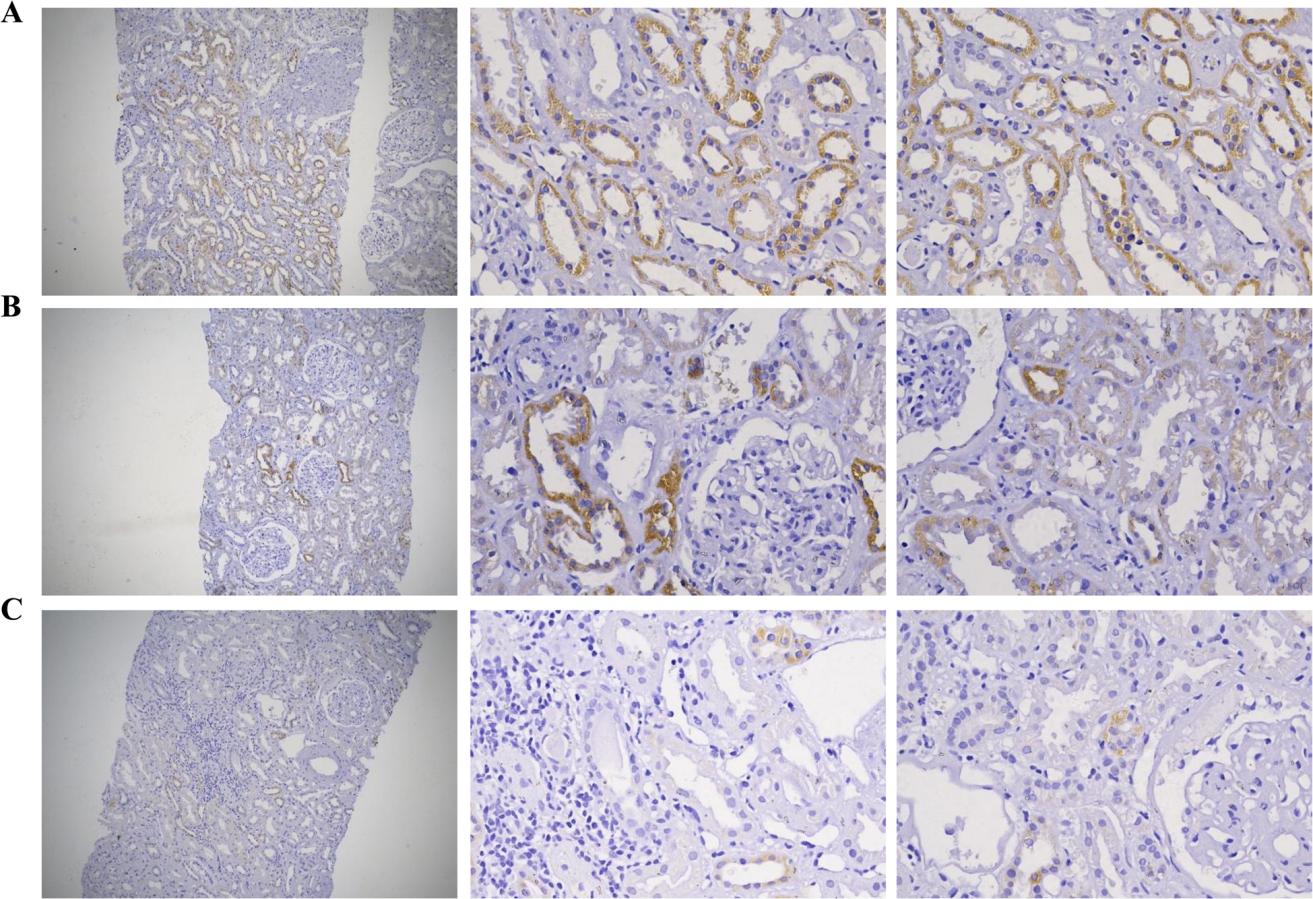
MPC2 expression in kidney tissues. Representative immunohistochemical staining of MPC2 in PGN (**a**, *n* = 30), PGN-DM (**b**, n = 30) and DN (**c**, *n* = 30) groups, respectively. Left column (Magnification× 100)*.* Right two columns (Magnification× 400)

**Fig. 3:**
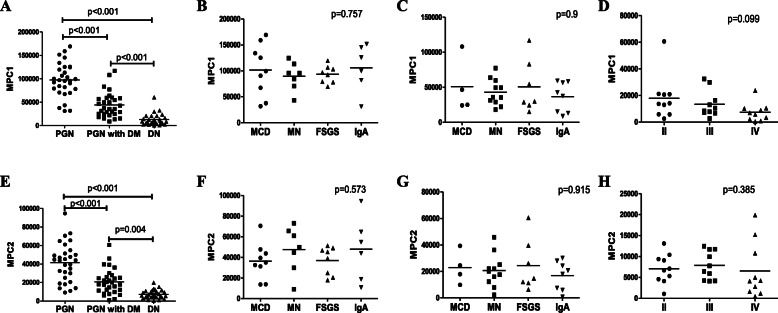
Quantitative analysis of MPC1 and MPC2 expressions. MPC1 expression in PGN, PGN-DM and DN groups (**a**). MPC1 expression in different types of pathology in the PGN group (**b**) and PGN-DM group (**c**). MPC1 expression among different pathological stages of DN (**d**). MPC2 expression in PGN, PGN-DM and DN groups (**e**). MPC2 expression in different types of pathology in the PGN group (**f**) and PGN-DM group (**g**). MPC2 expression among different pathological stages of DN (**h**)

Both MPC1 and MPC2 expression were confined to the cytoplasm of tubular cells. Typical cytoplasmic staining of MPC1 and MPC2 were shown in Fig. 1 and Fig. 2. Analysis of immunohistochemical staining showed that MPC1 expression was significantly lower in the DN group than the PGN-DM and the PGN group (both *p* < 0.001), and the PGN-DM group also had lower expression of MPC1 than the PGN group (*p* < 0.001) (Fig. 3a). The expression of MPC2 was consistent with MPC1 (Fig. 3e). No significant difference was observed in the expression of MPC1 and MPC2 among different pathological stages of DN (*p* = 0.099, *p* = 0.385, respectively) (Fig. 3d and h). Furthermore, we found no statistical differences of MPC1 and MPC2 expression in different types of pathology in the PGN-DM group (*p* = 0.9, *p* = 0.915, respectively) (Fig. 3c and g), and a similar trend was also noted in the PGN group (*p* = 0.757, *p* = 0.573, respectively) (Fig. 3b and f).

### Association between MPC and clinical parameters

**Fig. 4:**
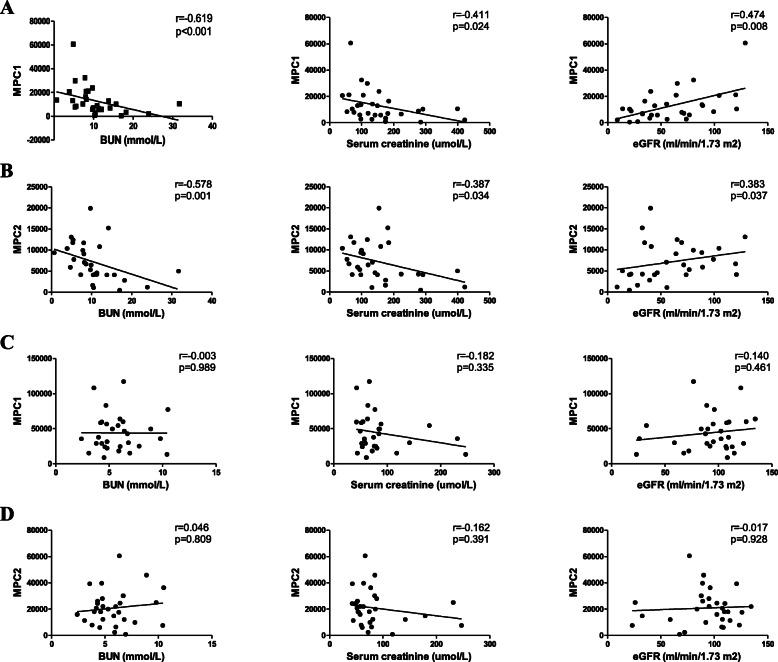
Association of MPC1 and MPC2 with renal functions. **a**: In DN group, the correlation between MPC1 and BUN, serum creatinine and eGFR, respectively. **b**: In DN group, the correlation between MPC2 and BUN, serum creatinine and eGFR, respectively. **c**: In PGN-DM group, the correlation between MPC1 and BUN, serum creatinine and eGFR, respectively. d: In PGN-DM group, the correlation between MPC2 and BUN, serum creatinine and eGFR, respectively

As shown in Table 2 and Fig. 4, in patients with diabetic nephropathy, the MPC1 was inversely correlated with serum creatinine (*r* = − 0.411, *p* = 0.024) and BUN (*r* = − 0.619, *p* < 0.001), whereas it was positively correlated with hemoglobin (*r* = 0.402, *p* = 0.028) and eGFR (*r* = 0.474, *p* = 0.008). And the MPC2 was negatively correlated with serum creatinine (*r* = − 0.387, *p* = 0.034) and BUN (*r* = − 0.578, *p* = 0.001), while it was positively correlated with HbA1c (*r* = 0.437, *p* = 0.029) and eGFR (*r* = 0.383, *p* = 0.037). There was no association between MPC and other clinical characteristics, including serum fasting glucose, cystatin C, urinary albumin, ACR, urinary titratable acid, and urinary ammonia, which showed significant differences among DN and control groups. Moreover, we assessed correlations of MPC expression and clinical features in the PGN-DM group. The negative correlation was found between MPC1 and urinary albumin (*r* = − 0.447, *p* = 0.013). And there were positive correlations between MPC2 and age (*r* = 0.485, *p* = 0.007), and HDL (*r* = 0.374, *p* = 0.042). There were no significant correlations between MPC and serum creatinine, BUN, and eGFR in the PGN-DM group, which showed significant correlations in the DN group.

**Table 2:** Associations between MPC and clinical features in DN and PGN-DM

	DN (*n* = 30)				PGN-DM (*n* = 30)			
	MPC1		MPC2		MPC1		MPC2	
	r	*p*	r	*p*	r	*p*	r	*p*
Gender	−0.106	0.578	−0.01	0.96	0.124	0.514	0.164	0.387
Age (years)	− 0.209	0.267	0.155	0.415	0.279	0.136	0.485	0.007
Diabetes duration (years)	0.085	0.655	0.123	0.517	− 0.088	0.646	0.1	0.601
Hypertension	−0.067	0.723	0.116	0.543	0.247	0.188	0.116	0.542
SBP (mmHg)	−0.111	0.559	0.052	0.784	0.159	0.401	0.264	0.158
DBP (mmHg)	−0.1	0.597	−0.053	0.781	−0.079	0.677	−0.124	0.515
Hb (g/L)	0.402	0.028	0.347	0.061	0.11	0.564	−0.143	0.452
HbA_1C_ (%)	0.375	0.065	0.437	0.029	0.16	0.436	0.089	0.664
Fasting glucose (mmol/L)	0.167	0.379	0.214	0.257	0.194	0.304	0.113	0.553
Total cholesterol (mg/dL)	−0.014	0.94	0.102	0.593	0.094	0.623	0.196	0.299
LDL cholesterol (mg/dL)	−0.058	0.762	0.104	0.585	0.09	0.635	0.136	0.475
HDL cholesterol (mg/dL)	0.09	0.636	0.002	0.992	0.323	0.082	0.374	0.042
Triglyceride (mg/dL)	0.031	0.871	0.153	0.421	−0.208	0.269	−0.306	0.1
Albumin (g/L)	0.081	0.669	0.035	0.854	0.256	0.173	0.21	0.265
Serum creatinine (umol/L)	−0.411	0.024	−0.387	0.034	−0.182	0.335	−0.162	0.391
eGFR (ml/min/1.73 m^2^)	0.474	0.008	0.383	0.037	0.14	0.461	−0.017	0.928
Serum cystatin C (mg/L)	−0.379	0.099	−0.4	0.081	−0.198	0.517	−0.082	0.789
BUN (mmol/L)	−0.619	< 0.001	−0.578	0.001	−0.003	0.989	0.046	0.809
Uric acid (umol/L)	−0.066	0.73	−0.288	0.123	−0.044	0.816	−0.202	0.284
Serum NGAL (ng/mL)	−0.33	0.107	−0.036	0.865	−0.343	0.128	−0.21	0.36
Urine NGAL (ng/mL)	−0.404	0.069	−0.113	0.626	−0.017	0.966	0.1	0.798
Urine albumin(g/24 h)	−0.282	0.131	−0.145	0.444	−0.447	0.013	−0.36	0.051
Urine ACR (mg/g)	−0.262	0.187	−0.343	0.08	−0.17	0.578	−0.165	0.59
Urine pH	−0.054	0.789	−0.211	0.29	−0.032	0.874	−0.206	0.302
Urine bicarbonate (mmol/L)	−0.151	0.452	−0.225	0.259	0.057	0.779	−0.09	0.656
Urine titratable acid (mmol/L)	0.052	0.797	0.223	0.264	0.249	0.21	0.205	0.306
Urine ammonia (mmol/L)	−0.06	0.765	−0.006	0.975	0.184	0.357	0.173	0.387

## Discussion

Mitochondrial dysfunction contributes to the development and progression of diabetic nephropathy, which is also related to renal injury in non-diabetic kidney diseases. However, the pathogenic mechanisms of mitochondrial dysfunction are still not incompletely understood in DN. In this study, we focused on associations between mitochondria-related MPC and kidney in diabetes and diabetic nephropathy. We found that both MPC1 and MPC2 expression in kidneys were significantly lower in the DN group than the PGN-DM and the PGN group, whereas there were no differences in MPC levels among DN stage II to stage IV. Moreover, both MPC1 and MPC2 levels were significantly correlated with serum creatinine, BUN and eGFR in patients with DN, whereas no analogous trend was observed in the non-diabetic kidney disease group.

The MPC is a gatekeeper in mitochondrial metabolism for transporting pyruvate, which is a central substrate in carbohydrate, fatty acid, and amino acid metabolism [19–22]. The MPC has been implicated in many diseases and exerted different expressions and effects in various disease models. Several studies have indicated that multiple cancers exhibited diminished MPC expression and activity, which causing dysfunction of mitochondrial pyruvate uptake. High glycolysis of tumor cells is known as the Warburg effect and the MPC is likely involved in the Warburg effect, which offers a potential target for cancer therapy [29–31]. Growing evidence supported that the altered mitochondrial function and the Warburg effect were closely correlated in the development of DN [32]. In our study, we found that MPC expression decreased in nondiabetic kidney disease coincident with diabetes mellitus and diabetic nephropathy, while diminished MPC expression was more serious in DN than nondiabetic kidney disease with DM. These findings were consistent with a recent study that the loss of renal MPC content was observed in db/db mice [33]. The mechanisms regulating MPC are incompletely understood. Thiazolidinediones, a widely used class of insulin sensitizers, were shown to bind to and inhibit the MPC [25]. The findings in liver, muscle and neurons have demonstrated that inhibition of MPC activities could be contextually beneficial in diseased conditions [34]. However, Feng et al. demonstrated that there was no different MPC expression in high glucose-treated podocytes compared to that in the control group, inhibition of MPC2 in podocytes led to pyruvate accumulation, mitochondrial dysfunction and cell apoptosis [35]. Regardless of loss in the expression or the activity of MPC, it all came with the reduced pyruvate transport, but whether this change was beneficial or pernicious might depend on types of disease. SIRT3, which is an NAD^+^-dependent deacetylase in mitochondria, targets several diverse enzymes involved in the regulation of the antioxidative defense system, metabolism and longevity [36]. SIRT3 activity corelated with the maintenance of mitochondrial energy homeostasis and antioxidant defense in proximal and distal tubule compartments [37]. The kidney of diabetic mice displayed time dependent reduction in the SIRT3 protein [38]. Also, high glucose decreased the protein and mRNA expression of SIRT3 in a time-dependent manner in cultured tubular epithelial cells [39]. A recent study showed that deacetylation modification by SIRT3 could influence MPC1 expression and activity likely via the inhibition of MPC1 degradation [40]. It is possible that the decrease of MPC might also be induced by SIRT3 reduction in diabetic renal tubules, which would partially explain why MPC was specifically reduced in DN with no difference in other glomerular kidney diseases. This study is an initial period for exploring changes of MPC in diabetic nephropathy, and further animal and cell researches are required.

Tubular damage occurs early in DN and may play a critical role in the progression of kidney disease [41]. Of note, renal tubules have a high density of mitochondria and increased glycolysis and glucose oxidation occur in tubules under hyperglycaemic conditions. In the setting of high glucose, tubular injury caused by mitochondrial dysfunction has been highlighted in the pathogenesis and development of DN [6]. Consistently, our present study showed that both MPC1 and MPC2 expressions were significantly lower in DN and correlated with serum creatinine, BUN and eGFR in patients with DN. Less clear is how decreased tubular MPC correlated with the glomerular injury. One explanation for this was reported by a previous study showing that proximal tubules could retrogradely interact with glomeruli and induce glomerular injury. However, there was no obvious association between renal MPC content and tubular marks as urinary NGAL, titratable acid, and ammonia in the present study. Although KIM-1 and NGAL are well-known tubular injury markers and previous study examined the expressions of tubular markers in DN and confirmed the associations between tubular expressions of NGAL and KIM-1 and renal function decline in DN, serum or urine NGAL levels as biomarkers is still limited since the NGAL protein, which would be affected by inflammation, is not specific to kidney injury [42]. Moreover, Mori K et al. presented a review that NGAL was a result of the active damage since atrophic nephrons did not produce NGAL [43]. Previous study reported that a sodium-glucose cotransporter-2 (SGLT-2) inhibitor, dapagliflozin, decreased urinary KIM-1 excretion, but did not affect urinary NGAL and LFAPB excretion. SGLT-2 expression was increased in diabetic patients and SGLT-2 inhibition reduced sodium and glucose reabsorption in the proximal tubule, thereby decreasing proximal tubular metabolic workload [44]. Further studies are warranted to determine whether the expression of MPC is correlated with other tubular injury markers.

MPC is fundamentally important in keeping glucose homeostasis. The roles of MPC in regulating glucose homeostasis are multivariate. MPC inhibition in the pancreas contributed to glucose intolerant, while inhibition of the MPC in muscle or liver might ameliorate glucose tolerance [45]. The kidney is an important player in regulating glucose homeostasis through its utilization, gluconeogenesis, and reabsorption in the proximal renal tubule via sodium glucose cotransporters [46]. However, no notable association between MPC expression and glucose was observed in this study. The roles of decreased MPC expression in DN for glucose homeostasis are poorly understood.

This was the first study validating the loss of MPC in the renal tissue of DM and DN, and illustrating obvious correlations between MPC and renal function in DN. The major limitation of the present study was that the lack of insufficient indicators to test pyruvate and mitochondrial function and changes in the MPC-pyruvate-mitochondria axis remained unclear. Moreover, the causal relationship and mechanism between MPC disruption and DN were not explored. Further studies will be required to elucidate the precise mechanisms.

## Conclusion

In summary, decreased MPC content was more pronounced in diabetic nephropathy than nondiabetic kidney disease. Furthermore, MPC levels were significantly correlated with renal functions in patients with DN. Further studies are warranted to determine whether MPC would be a therapeutic target in DN.

## Data Availability

The datasets analyzed during the current study are available from the corresponding author on reasonable request.

## Abbreviations

DN: Diabetic nephropathy
MPC1: Mitochondrial pyruvate carrier 1
MPC2: Mitochondrial pyruvate carrier 2
PGN: Primary glomerulonephropathy
DM: Diabetes mellitus
MN: Membranous nephropathy
IgAN: IgA nephropathy
FSGS: focal segmental glomerulosclerosis
MCD: Minimal change disease
SBP: Systolic blood pressure
DBP: Diastolic blood pressure
HbA_1C_: Glycosylated hemoglobin
BUN: Blood urea nitrogen
LDL: Low density lipoprotein
HDL: High density lipoprotein
NGAL: Neutrophil gelatinase-associated lipocalin
ACR: Albumin-to-creatinine ratio
eGFR: Estimated glomerular filtration rate
CKD-EPI: Chronic kidney disease epidemiology collaboration
SGLT-2: Sodium-glucose cotransporter-2
